# A Core Genome Multilocus Sequence Typing Scheme for Streptococcus mutans

**DOI:** 10.1128/mSphere.00348-20

**Published:** 2020-07-08

**Authors:** Shanshan Liu, Xiaoliang Li, Zhenfei Guo, Hongsheng Liu, Yu Sun, Yudong Liu, Qinglong Wang, Shengkai Liao, Kai Zhang

**Affiliations:** a Department of Stomatology, The First Affiliated Hospital of Bengbu Medical College, Bengbu, China; b Department of Stomatology, Bengbu Medical College, Bengbu, China; c Department of Biochemistry and Molecular Biology, Bengbu Medical College, Bengbu, China; d Department of histology and Embryology, Bengbu Medical College, Bengbu, China; University of California, Davis

**Keywords:** molecular biology, *Streptococcus mutans*, cgMLST, whole-genome sequence

## Abstract

Streptococcus mutans is one of the primary pathogens responsible for the development of dental caries. Recent whole-genome sequencing (WGS)-based core genome multilocus sequence typing (cgMLST) approaches have been employed in epidemiological studies of specific human pathogens. However, this approach has not been reported in studies of S. mutans. Here, we therefore developed a cgMLST scheme for S. mutans. We surveyed 199 available S. mutans genomes as a means of identifying cgMLST targets, developing a scheme that incorporated 594 targets from the S. mutans UA159 reference genome. Sixty-eight sequence types (STs) were identified in this cgMLST scheme (cgSTs) in 80 S. mutans isolates from 40 children that were sequenced in this study, compared to 35 STs identified by multilocus sequence typing (MLST). Fifty-six cgSTs (82.35%) were associated with a single isolate based on our cgMLST scheme, which is significantly higher than in the MLST scheme (11.43%). In addition, 58.06% of all MLST profiles with ≥2 isolates were further differentiated by our cgMLST scheme. Topological analyses of the maximum likelihood phylogenetic trees revealed that our cgMLST scheme was more reliable than the MLST scheme. A minimum spanning tree of 145 S. mutans isolates from 10 countries developed based upon the cgMLST scheme highlighted the diverse population structure of S. mutans. This cgMLST scheme thus offers a new molecular typing method suitable for evaluating the epidemiological distribution of this pathogen and has the potential to serve as a benchmark for future global studies of the epidemiological nature of dental caries.

**IMPORTANCE** Streptococcus mutans is regarded as a major pathogen responsible for the onset of dental caries. S. mutans can transmit among people, especially within families. In this study, we established a new epidemiological approach to S. mutans classification. This approach can effectively differentiate among closely related isolates and offers superior reliability relative to that of the traditional MLST molecular typing method. As such, it has the potential to better support effective public health strategies centered around this bacterium that are aimed at preventing and treating dental caries.

## Abstract (precis)

Streptococcus mutans is regarded as a major pathogen responsible for the onset of dental caries. S. mutans can transmit among people, especially within families. In this study, we established a new epidemiological approach to S. mutans classification. This approach can effectively differentiate among closely related isolates and offers superior reliability relative to that of the traditional MLST molecular typing method. As such, it has the potential to better support effective public health strategies centered around this bacterium that are aimed at preventing and treating dental caries.

## INTRODUCTION

Dental caries are highly prevalent bacterial infections in humans, and they can have a significant adverse impact on the mental and physical health of affected individuals (1–3). One of the primary drivers of these caries is the Gram-positive bacterium Streptococcus mutans, as its colonization of the oral cavity is closely linked to caries initiation and progression (4). S. mutans bacteria are able to readily adhere to the surface of a tooth, forming a biofilm and releasing acidic compounds following carbohydrate metabolism, thereby corroding the tooth surface (5). There is strong epidemiological evidence that S. mutans is more commonly detected in the oral cavity of children affected by dental caries compared to that in children free of such caries (6, 7), and the forms of S. mutans isolated from children with caries have been found to be more virulent than those isolated from caries-free children (8). There is thus a clear need to better understand the genetic diversity of S. mutans in order to more fully elucidate its pathogenicity and its ability to be transmitted between humans.

S. mutans has previously been differentiated by serotyping (*c*, *e*, *f*, and *k*) on the basis of the composition of its cell surface rhamnose-glucose polymers (9, 10). However, this approach makes it difficult to separate strains of the same serotype. Genotypic methodologies, including ribotyping, arbitrary primed PCR (AP-PCR), chromosomal DNA restriction fragment polymorphism, repetitive extragenic palindromic PCR (rep-PCR), multilocus enzyme electrophoresis, and pulse-field gel electrophoresis (PFEG), have been used to subtype S. mutans (11–16). These phenotypic approaches, however, are time intensive and have difficulty differentiating between closely related bacterial strains (17). In 2007, Nakano et al. first reported the use of multilocus sequence typing (MLST) in order to more effectively and reliably discriminate between S. mutans strains (17). MLST strategies allow for an efficient analysis of the relationship between bacterial strains by sequencing defined conserved housekeeping genes and comparing this genotypic information between isolates (17). This approach can more reliably and reproducibly detect differences between closely related bacterial strains, as traditional gel-based typing strategies are based upon subjective similarity, whereas single-nucleotide mutations can be detected via MLST, thus allowing for the identification of a new sequence type (ST). Another MLST scheme was also reported by Do et al. in 2010 (18).

In recent years, a core genome MLST (cgMLST) strategy has been used to differentiate between bacterial strains with even greater reliability than traditional MLST approaches, as cgMLST strategies incorporate more sequence data than the 5 to 8 housekeeping genes typically sequenced for MLST (19, 20). Such cgMLST approaches have been successfully used to type many pathogens, including Mycoplasma synoviae, Brucella spp., Yersinia, Listeria monocytogenes, Staphylococcus capitis, Staphylococcus argenteus, and Staphylococcus aureus (21–27). While such cgMLST strategies have been well documented, they have not been well studied with respect to S. mutans. As such, in the present study, we defined a S. mutans cgMLST scheme and compared the resolution between MLST and cgMLST schemes.

## RESULTS

### Candidate cgMLST target gene set.

We began by identifying genes that were well-suited to incorporation into our cgMLST scheme by filtering the S. mutans UA159 (GenBank accession number NC_004350.2) reference genome based upon minimum gene length, gene overlap, and start/stop codon positioning. This led to the identification of 1,811 genes, and this number was further winnowed to 1,797 genes following the removal of plasmid and transposon genes. These genes were then retained as candidate targets for cgMLST analyses.

### Candidate genomes.

**FIG 1:**
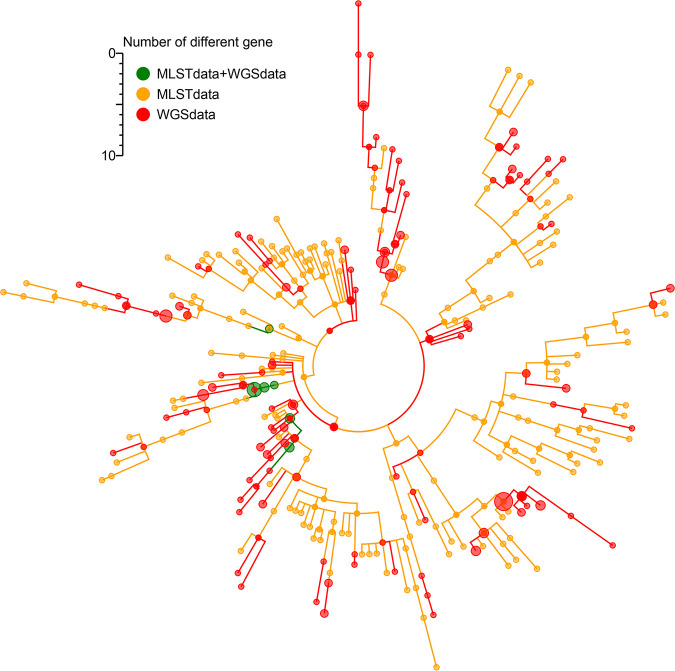
A representation of 161 candidate S. mutans genomes based upon the S. mutans multilocus sequence typing (MLST) database. Sequences corresponding to the eight housekeeping genes that comprise the allelic profile of the Nakano MLST scheme were extracted from these 161 genomes and were queried against the S. mutans Nakano MLST database. In the resultant minimum spanning tree, these 161 genomes (whole-genome sequencing [WGS] data) were found to be distributed throughout the MLST data, indicating that this 161-genome data set corresponds to a satisfactory representation of the S. mutans population.

We downloaded 199 total S. mutans genomes from the National Center for Biotechnology Information (NCBI) database for screening purposes. Of these genomes, 33 that lacked the eight traditional MLST gene segments were eliminated from further analysis, as were 5 genomes in which single copy gene numbers were <75%. The remaining 161 genomes were then used as candidates to screen our candidate cgMLST target genes. We additionally extracted the sequences corresponding to the eight housekeeping genes traditionally used for MLST-based bacterial typing, and we compared these sequences to the S. mutans MLST database. We then constructed a minimum spanning tree based upon the allelic profiles of these 161 genomes, the 161 genomes distributed in the whole tree, revealing them to be well distributed throughout the tree (Fig. 1; see also Table S1 in the supplemental material).

10.1128/mSphere.00348-20.3 TABLE S1 The 161 candidate genome identifiers (IDs). Download Table S1, XLSX file, 0.01 MB. Copyright © 2020 Liu et al. 2020 Liu et al. This content is distributed under the terms of the Creative Commons Attribution 4.0 International license.

### cgMLST target gene definition, evaluation, and potential functional characteristics.

**FIG 2:**
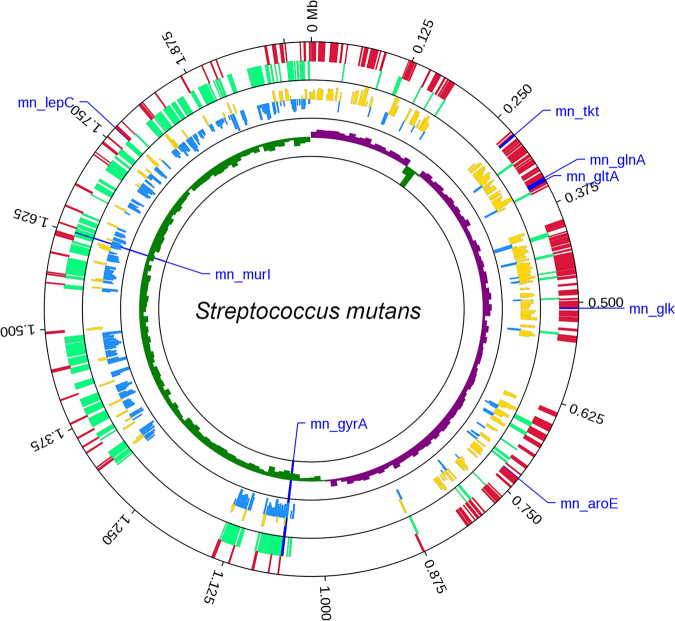
Distribution of the 594 cgMLST target genes within the S. mutans UA159 reference genome. The eight reference genes used for standard MLST genes are noted. Target genes are shown in the outer circle, with green and red corresponding to those genes on the negative and positive strands, respectively. The allele numbers for cgMLST target genes are shown in the middle circle, with blue and yellow corresponding to genes on the negative and positive strands, respectively. The GC skew value [the ratio of (G − C)/(G + C)] is also shown, with green and violet corresponding to these skew values on the negative and positive strands, respectively. The S. mutans UA159 genome (GenBank assembly number GCA_000007465.2) served as a reference for this analysis.

Using these 161 genomes, we next sought to better define the list of target genes suitable for inclusion in our cgMLST scheme. Based on the criteria defined in Materials and Methods, we were able to identify 594 core genes (30.3% of the reference genome) suitable for incorporation into our cgMLST scheme (Fig. 2; see also Table S2 in the supplemental material). To preliminarily explore the potential functional characteristics of these target genes, gene ontology (GO) terms and Kyoto Encyclopedia of Genes and Genomes (KEGG) pathways in which they were enriched were identified using the Database for Annotation, Visualization and Integrated Discovery (DAVID) software (see Fig. S1 in the supplemental material). We found that the target genes were associated with translation and ATP binding capacity. A total of 8 enriched pathways were identified via KEGG analysis as being significantly enriched for these genes (*P < *0.05), including the pathways for carbon metabolism, glycolysis, biosynthesis of secondary metabolites crucial to acid production, cell growth, and antagonism of other oral streptococci in S. mutans.

10.1128/mSphere.00348-20.1 FIG S1 Gene ontology (GO) and Kyoto Encyclopedia of Genes and Genomes (KEGG) enrichment analyses of core genome multilocus sequence typing (cgMLST) scheme target genes. (A to C) GO terms identified via a functional enrichment analysis associated with biological processes, cellular components, and molecular functions, with a *P* value of <0.05 as a cutoff for significance, and (D) biological pathways identified via a KEGG analysis with a *P* value of <0.05 as a cutoff for significance. Download FIG S1, TIF file, 1.4 MB. Copyright © 2020 Liu et al. 2020 Liu et al. This content is distributed under the terms of the Creative Commons Attribution 4.0 International license.

10.1128/mSphere.00348-20.4 TABLE S2 The 597 core genes in the cgMLST scheme. Download Table S2, XLSX file, 0.02 MB. Copyright © 2020 Liu et al. 2020 Liu et al. This content is distributed under the terms of the Creative Commons Attribution 4.0 International license.

### Comparison between MLST and cgMLST schemes.

Based on the MLST and cgMLST schemes, the sequenced isolates were found to belong to 35 distinct STs and 68 distinct core genome sequence types (cgSTs), respectively. According to the MLST scheme, a total of 4 STs (ST234, ST249, ST265, and ST269) were associated with a single isolate, accounting for 11.43% of the 35 STs. However, there were 56 cgSTs (82.35%) that were associated with a single isolate according to the cgMLST scheme, with this frequency being significantly higher than that in the MLST scheme (*P < *0.001). Although some of these isolates were still found to be identical using this cgMLST scheme, 58.06% of all MLST profiles that contained ≥2 isolates were further differentiated by our cgMLST scheme (18 out of 31 STs). For example, the SMB6, SMB37, SMB79, and SMB80 isolates shared an identical ST (ST236) based upon the MLST scheme, whereas these ST236 isolates were separated into different cgSTs (cgST56, cgST29, cgST68, and cgST67 for SMB6, SMB37, SMB79, and SMB80, respectively) using our S. mutans cgMLST scheme. Each child provided two isolates. The two isolates from 70% of these children (28 out of the 40) were distinguished using our cgMLST scheme, with this rate being significantly higher than that achieved when using the MLST scheme, which yield a 7.5% discrimination rate (*P < *0.001).

**FIG 3:**
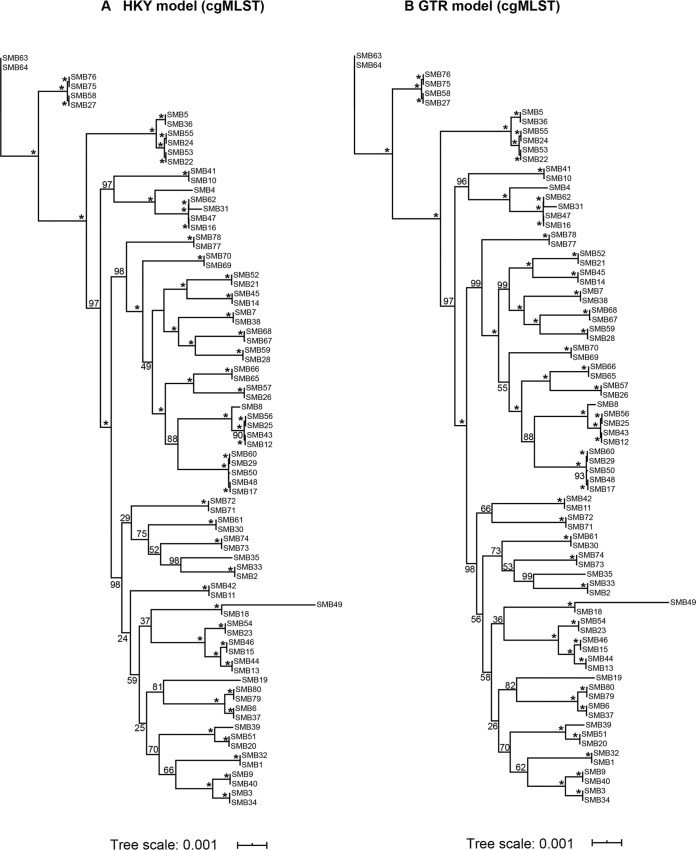
Phylogenetic trees corresponding to the 80 sequenced samples constructed according to the cgMLST scheme. Trees were generated using the maximum likelihood method. (A) Tree constructed under the HKY model and (B) tree constructed with the GTR model. Numbers on lines indicate bootstrap values determined for 1,000 replicates. Scale bar indicates the proportion of nucleotide substitutions. An asterisk (*) indicates 100%.

**FIG 4:**
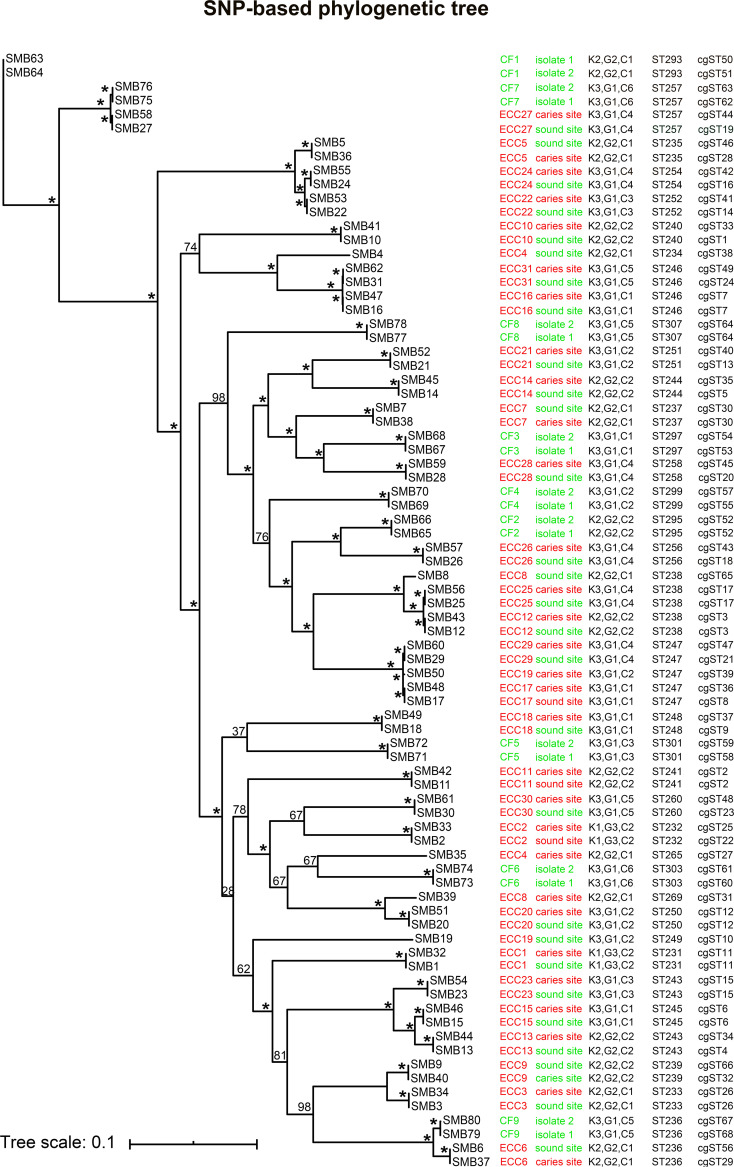
Single-nucleotide polymorphism (SNP)-based phylogenetic trees generated using the maximum-likelihood method under an HKY model. Numbers on lines are bootstrap values determined for 1,000 replicates. An asterisk (*) corresponds to 100%. CF, caries free; ECC, early childhood caries. Each child provided two isolates. CF1 to CF9 represent the identifiers (IDs) for CF children. ECC1 to ECC31 represent the IDs of children with ECC. CF children provided two isolates from the surfaces of healthy teeth, while ECC children provided one isolate from a sound site and one isolate from a caries site. CF children and their isolates are marked in green, whereas ECC children and their isolates from caries sites are marked in red and ECC child isolates from sound sites are marked in green. K, G, and C correspond to kindergarten, grade, and class, respectively.

The topologies of the phylogenetic trees developed using Hasegawa-Kishino-Yano (HKY) and general time-reversible (GTR) models were highly congruent in the cgMLST scheme (Fig. 3). Both of the trees indicated that these isolates may have originated from SMB63 and SMB64. Strong bootstrapping support was observed in both trees. However, the topologies of the two trees developed when using the MLST scheme were relatively lower and they exhibited reduced bootstrap support (see Fig. S2 in the supplemental material). The topologies of the cgMLST scheme-based phylogenetic tree and a single-nucleotide polymorphism (SNP)-based phylogenetic tree generated under the HKY model were more congruent, whereas the topological structural consistency of the phylogenetic trees based upon the cgMLST scheme and the SNP-based approach generated under the HKY model was relatively poor (Fig. 4).

10.1128/mSphere.00348-20.2 FIG S2 Maximum likelihood phylogenetic trees corresponding to the 80 sequenced samples based upon the MLST scheme. (A) Tree constructed under the HKY model and (B) tree constructed with the GTR model. Numbers on lines indicate bootstrap values determined for 1,000 replicates. Scale bar indicates the proportion of nucleotide substitutions. For easy visualization, only the bootstrap values above 70% are marked with an asterisk (*). Download FIG S2, TIF file, 1.1 MB. Copyright © 2020 Liu et al. 2020 Liu et al. This content is distributed under the terms of the Creative Commons Attribution 4.0 International license.

### S. mutans epidemiological analysis.

**FIG 5:**
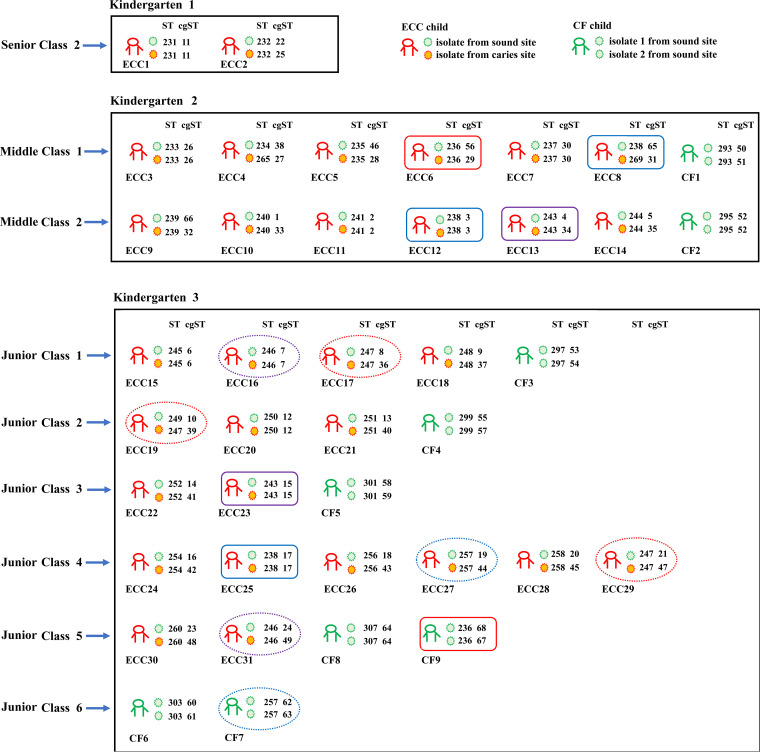
Epidemiological analysis of the 80 S. mutans isolates from Bengbu, China. CF children and isolates are marked in green, whereas ECC children and the isolates from caries site are marked in red and isolates from the sound sites in these ECC children are marked in green. Square boxes indicate transmission between kindergartens, and ovals indicate transmission between different classes within a given kindergarten. Isolates with identical STs according to the MLST scheme are marked in the same color using square boxes or ovals.

**FIG 6:**
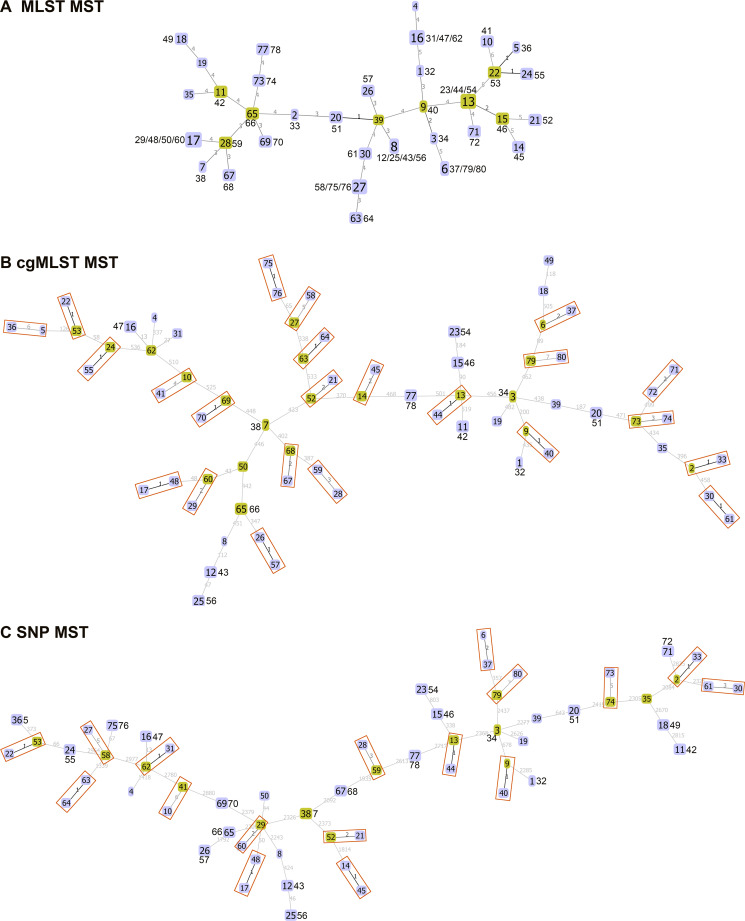
Minimum spanning trees (MST) of the 80 S. mutans isolates sequenced in this study. (A) MST based on MLST, (B) MST based on cgMLST, and (C) MST based on core genome SNPs. Numbers in boxes correspond to the SMB ID (SMB1 to SMB80). A yellow background indicates samples in nodes, and blue backgrounds indicate samples in branches. Red squares represent the samples with identical STs according to the MLST scheme that were further distinguished by cgMLST or by core genome SNPs. Numbers on lines correspond to the number of different alleles in MLST and cgMLST and the number of SNP differences in the core genome.

**FIG 7:**
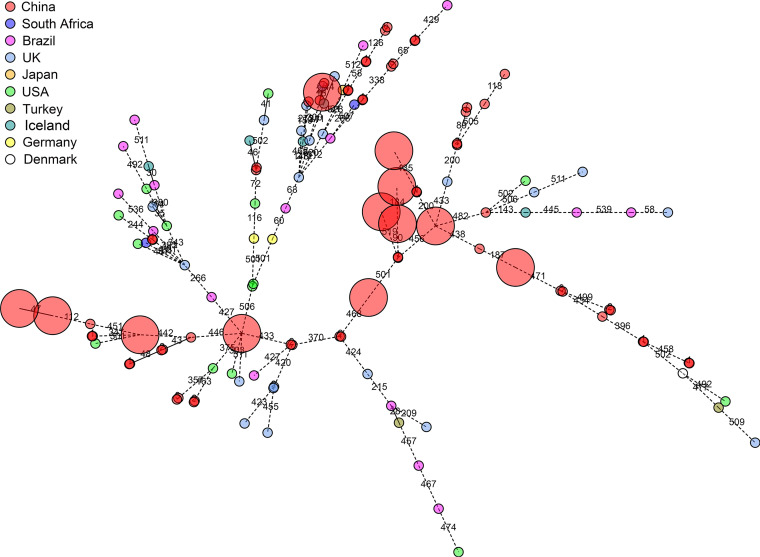
Minimum spanning tree analysis of the cgMLST profiles of 145 S. mutans isolates from 10 countries. The 80 different S. mutans isolates from China were sequenced specifically for the present study, while the whole genome sequences of the other 65 international isolates were downloaded from the NCBI database. Circles correspond to the allelic profiles of individual strains based upon analyses of cgMLST target gene sequences. Numbers on lines correspond to the number of target genes for which allelic differences were detected. Circle colors are used to differentiate the country of origin of the isolates. The core genome sequence types (cgSTs) at the nodes were removed for easy visualization. Isolates with identical cgSTs that originated from China are shown with large red circles.

Child-to-child transmission of S. mutans between different kindergartens and between different classes in the same kindergarten was found to have occurred in Bengbu, China, according to the MLST scheme (Fig. 5). Among the 40 pairs of S. mutans isolates obtained from a given child, 37 pairs of isolates shared the same ST according to this MLST scheme, suggesting them to be epidemiologically related isolates. Among these 37 pairs of isolates, there were 12, 20, and 8 pairs of isolates that shared the same cgST, core genome SNPs, and total SNPs, respectively. More than 92% of epidemiologically related pairs of isolates were differentiated based upon 7 differences in core genome SNPs, 10 differences in total SNPs, and 7 alleles in cgMLST scheme. Minimum spanning trees constructed based on MLST, cgMLST, and core genome SNP analyses of these 80 isolates are shown in Fig. 6. A minimum spanning tree constructed based on the resultant cgMLST analysis using 145 isolates from 10 countries showed that no close epidemiological relationships among isolates were observed among different countries (≤7 allele differences) (Fig. 7). For details regarding the number of allele differences among these isolates, see Table S3 in the supplemental material.

10.1128/mSphere.00348-20.5 TABLE S3 Numbers of differing alleles in these 145 isolates. Download Table S3, XLSX file, 0.02 MB. Copyright © 2020 Liu et al. 2020 Liu et al. This content is distributed under the terms of the Creative Commons Attribution 4.0 International license.

## DISCUSSION

In this study, we observed the distribution of 161 candidate S. mutans genomes throughout the tree, consistent with these genomes being representative of the diversity of S. mutans populations (Fig. 1). In prior studies, the proportion of core genes from the reference genome has ranged from 13.3% to 59.33% (28–31). In the present analysis, we retained 594 core target genes in our cgMLST scheme, corresponding to 30.3% of the reference genome. Differences in the proportion of candidate genomes included in each study influence the number and thus the percentage of core genes. For example, a total of 634 cgMLST (13.3% of the reference genome) targets were found based on 167 genomes for Klebsiella pneumoniae, whereas the number of targets rose to 1143 (24.0%) when the number of genomes increased to 907 (30, 31). In addition, the genome length may also influence the number and the percentage of core genes, with longer genomes including a greater proportion of target genes in the genome. For example, the median total length of the Listeria monocytogenes, Mycoplasma gallisepticum, and S. mutans genomes retrieved from the NCBI database (14 February 2020) were 2.97 Mb, 0.96 Mb, and 1.96 Mb, respectively. In contrast, the median total length of the K. pneumoniae genome is 5.59 Mb, and its core target genes account for just 24.0% of its reference genome. Therefore, the size of the genome is not the sole factor that determines the number of target genes. In the MLST scheme, housekeeping genes were evenly spaced throughout the reference genome, as this is critical for such schemes (18). Similarly, the target genes in this study were distributed throughout the genome (Fig. 2), confirming the validity and utility of this core gene set (30).

GO annotation and KEGG analyses revealed that these target genes were involved in 8 pathways and 5 molecular functions (*P < *0.05) that may be related to cell growth under different conditions and to cariogenicity. For example, in S. mutans, the l-lactate dehydrogenase, enzyme encoded by *ldh* (SMU_1115) and activated by fructose-1,6-diphosphate (FDP), can catalyze the conversion of pyruvate to lactic acid and decrease the pH of the growth medium during glycolysis (32–34). The low-pH environment, in turn, leads to the demineralization of the tooth surface. Mutacin IV is an effective bacteriocin produced by S. mutans that can antagonize the growth of several oral streptococcal species (35). Mutacin IV is a core secondary metabolite according to the antiSMASH analysis presented in our previous study (36). SepM, a membrane-associated polypeptide encoded by *sepM* (SMU_518) in S. mutans, is a prerequisite for the synthesis of mutacin IV (37).

As shown in a previous study, 47 S. mutans isolates were analyzed with MLST scheme resulting in 29 STs, compared with 22 rep-PCR genotypes (38). Seven subtypes were further differentiated by MLST. In the present study, more cgSTs (*n* = 68) were defined based upon our cgMLST scheme relative to the 35 STs defined by this prior MLST scheme, and 82.35% of cgSTs were associated with a single isolate among these 68 STs, which is a significantly higher percentage than that under the MLST scheme. In addition, 58.06% of the MLST profiles associated with ≥2 isolates were further differentiated using our cgMLST scheme (18/31 STs). Each child in the present study provided two isolates, and in 70% of children, these two isolates were distinguished by our cgMLST scheme, with this rate being significantly higher than that achieved with the MLST scheme (7.5%). The topologies of the phylogenetic trees generated under HKY and GTR models were highly congruent and exhibited strong bootstrap support when using our cgMLST scheme, in contrast to the trees derived from the MLST scheme. These data suggest that this S. mutans cgMLST scheme is of higher reproducibility than the MLST scheme in question. The consistency of the topological structure of the phylogenetic tree constructed using our cgMLST scheme and SNP methods was better than that of the phylogenetic tree constructed using the MLST scheme and SNP methods. This indicates the higher reliability of our cgMLST scheme relative to that of this MLST scheme.

Reported patterns of S. mutans transmission include mother-to-child transmission, father-to-child transmission, intrafamilial transmission, and child-to-child transmission (39–41). In previous studies of S. mutans, evidence of transmission among children and their family members was defined via MLST based on identical ST assignments (17, 40). In this study, child-to-child transmission of S. mutans between different kindergartens and between different classes in the same kindergarten was observed in Bengbu, China, according to the MLST scheme. However, based upon our cgMLST scheme, 25 pairs of isolates out of 37 pairs of isolates that had shared identical ST assignments were differentiated by cgST, with our cgMLST scheme having resulted in a larger genetic distance between isolates than the MLST scheme for the majority of isolate comparisons. This emphasized the higher discriminatory power of our cgMLST scheme relative to that of the MLST scheme, suggesting that the differences in the cgMLST target genes observed pairs of isolates from a given child may be the result of intrahost and/or intraniche evolution. In addition, this indicates that our cgMLST scheme is superior to the MLST scheme for the molecular typing of closely epidemiologically related isolates. In the absence of a gold standard molecular typing method with perfect discriminatory power, epidemiological link data were used to define related and unrelated isolates, with maximal contrast between related and unrelated isolates being sought. Epidemiologically related isolates were defined as being obtained from the same patient, belonging to the same ST, and cultured within a time window of 30 days (42). The threshold for cluster types was identified when 92% of all likely epidemiologically related pairs of isolates were differentiated (43). We utilized these criteria as a reference to identify the threshold for SNP and allele differences in cgSTs in the current study. We found that 92%, 100%, and 97% of epidemiologically related pairs of isolates differed by 7 alleles in our cgMLST scheme, 7 core genome SNPs, and 10 total SNPs, respectively. We combined 65 previously published sequences and 80 freshly prepared S. mutans sequences to analyze the diverse population structure of S. mutans (44, 45), and we found that there are 37 pairs of isolates that shared ≤7 allele differences and that were thus considered to be a clonally related based on our cgMLST scheme. Among these, 1 pair of isolates originated from the United Kingdom, 1 pair of isolates originated from the United States, and the remaining 35 pairs of isolates originated from China. No close epidemiological links among isolates were observed among different countries, with the fewest allele differences among countries being observed for isolates from China and the United Kingdom (22 alleles). These differences thus reveal that the genetic diversity of S. mutans is complex.

Although our study provides a new molecular typing method for the epidemiologic analysis of S. mutans, it does have some limitations. More than half of the STs were differentiated by cgST, but there were still 12 pairs of isolates were indistinguishable via this approach. Given that the number of candidate genomes influences the percentage of core genes in a cgMLST scheme, the use of more samples to prepare a cgMLST scheme for S. mutans may improve this scheme and make it more practical, particularly as a means of differentiating between closely related isolates. Moreover, the small number of samples of the S. mutans isolates from several countries used in this study (1 from Denmark, 2 from Germany, 1 from Japan, 2 from South Africa, and 2 from Turkey) may interfere with the results of our epidemiological analysis.

### Conclusion.

In summary, our cgMLST scheme represents a powerful and effective tool for the epidemiological typing of S. mutans isolates. By leveraging this tool, it will be possible to better understand S. mutans population structures and dynamics, thus allowing more effective development of public health strategies centered around this bacterium.

## MATERIALS AND METHODS

### Construction of a primitive cgMLST target gene set.

For this study, the S. mutans UA159 (GenBank assembly accession number GCA_000007465.2) reference genome was used to guide analysis and candidate target gene identification. Candidate genes for the cgMLST scheme were selected by discarding genes that met the following criteria using balstall 2.2.26: genes that were <50 bp, genes lacking a start or stop codon; genes with multiple stop codons or with nonsense mutations resulting in premature stop codon positioning, the shorter of any pairs of genes that overlapped by >4 bp, genes with high homology to the transposon_db TransposonPSI database (http://transposonpsi.sourceforge.net/; accessed September 2017) (identity > 50%, coverage > 70%), and/or genes with high homology with plasmid genomes (ftp://ftp.ncbi.nlm.nih.gov/genomes/refseq/plasmid/; accessed April 2018) (identity > 90%, overlap > 95%) (30).

### Candidate genome set construction.

We identified 199 total S. mutans whole genomes that were available from the National Center for Biotechnology Information (NCBI) as of 1 December 2019. Genomes were filtered based on the following criteria via blastall 2.2.26: genomes with a contig number of ≥200 and a scaffold of <500 bp, genomes not containing all MLST core genes, and/or genomes having a single gene copy number of <75% (30). Both Nakano’s and Do’s MLST approaches demonstrate similar discrimination for S. mutans (46). However, only two candidate genomes were preserved after data filtering based on Do’s MLST scheme. Therefore, only Nakano’s MLST scheme was used in this study. Information regarding the resultant 161 candidate genomes is listed in Table S1 in the supplemental material.

### cgMLST target gene identification and functional annotation.

The following criteria were used to define the target genes used in our cgMLST scheme via blastall 2.2.26: keep all single copy genes identified by a BLAST search in both candidate gene set and genomes and filter the genes of the primitive gene set with default parameter of stop codon or genes that have internal stop codons in more than 20% of the candidate genomes (29, 47). Information pertaining to the resultant 594 cgMLST target genes is listed in Table S2 in the supplemental material. All candidate genomes were then typed based upon the cgMLST target genes, and these genomes were integrated with STs defined in the MLST database (29 August 2019) (https://pubmlst.org/oralstrep/) in order to conduct a minimum spanning tree analysis. We evaluated these cgMLST target genes based upon candidate genome coverage and the distribution of cgMLST target genes in the genome of S. mutans UA159. The Database for Annotation, Visualization and Integrated Discovery (DAVID 6.8) gene annotation tool (https://david.ncifcrf.gov/) was employed for functional annotation bioinformatics microarray analysis, including Kyoto Encyclopedia of Genes and Genomes (KEGG) pathways and gene ontology (GO) associated with target genes in this cgMLST scheme (48). All of the pathways or functional categories meeting the following cutoff criterion were extracted for analysis: *P* < 0.05.

### Whole-genome sequencing and assembly.

For this study, the whole genomes of 80 S. mutans isolates that were obtained from the supragingival dental plaque of children in Bengbu, China, were preserved in our laboratory and were sequenced and assembled (see Table S4 in the supplemental material). A TIANamp bacteria DNA kit (Tiangen Biotech, Beijing, China) was used for bacterial genomic DNA (gDNA) isolation based on provided directions, after which 1 μg of DNA from each sample was used with a NEBNext Ultra DNA library prep kit for Illumina (NEB, USA) in order to prepare libraries for sequencing. Barcodes were added to each sample during this process. Samples were then sonicated into 350-bp fragments, followed by end polishing, poly(A) tail processing, ligation to full-length Illumina adaptors, and additional PCR amplification. The AMPure XP system was then used to extract the amplified libraries, which were subjected to quality control analysis and quantification using an Agilent 2100 Bioanalyzer and via real-time PCR. An Illumina NovaSeq 6000 PE150 instrument was then used to sequence these 80 whole genomes at Novogene Bioinformatics Technology (Beijing, China), and the SOAPdenovo software was used for sequence assembly.

10.1128/mSphere.00348-20.6 TABLE S4 Characteristics of Streptococcus mutans isolates sequenced in this study. Download Table S4, XLSX file, 0.01 MB. Copyright © 2020 Liu et al. 2020 Liu et al. This content is distributed under the terms of the Creative Commons Attribution 4.0 International license.

### Comparison between MLST and cgMLST.

A total of 80 sequenced samples were used to assess the resolution of this S. mutans cgMLST scheme by comparing the cgMLST-ST (cgST) and Nakano MLST-ST (ST). The numbers of STs and cgSTs were analyzed using a Pearson Chi-square test by SPSS 20.0 software (IBM, Armonk, NY, USA). A *P* value of <0.05 was considered statistically significant. Maximum likelihood phylogenetic trees were generated based on 594 cgMLST core genes and 8 MLST housekeeping genes in all 80 samples via TreeBeST under Hasegawa-Kishino-Yano (HKY) and general time-reversible (GTR) models (49–51). The reliability of the branching orders was evaluated via 1,000 bootstrap replicates (52). The Phylogenetic trees were annotated using Interactive Tree Of Life (iTOL) v5 (https://itol.embl.de/) (53). The allele profiles and cgSTs of the 199 genomes downloaded from the NCBI database and of the 80 isolates sequenced in this study are listed in Tables S5 and S6 in the supplemental material.

10.1128/mSphere.00348-20.7 TABLE S5 Allele profiles based on the core genes of our cgMLST scheme. Download Table S5, XLSX file, 0.6 MB. Copyright © 2020 Liu et al. 2020 Liu et al. This content is distributed under the terms of the Creative Commons Attribution 4.0 International license.

### Whole-genome sequence analysis for SNP-based phylogeny.

Each sample was compared with the reference sequence to identify the potential SNP sites using MUMmer 3.23 (54); 100-bp sequences on both sides of the reference sequence SNP sites were extracted, and then the extracted reference sequence and the assembly results were compared to verify the SNP sites. If the alignment length was less than 101 bp, it was considered to be an untrustworthy SNP and was removed. Tandem Repeats Finder 4.07b and RepeatMasker 4.0.5 were used to evaluate the repeated sequence area of the reference sequence and to filter the SNPs located in the repeated area. Finally, a reliable SNP was obtained. SNP-based phylogenetic trees were built using TreeBeST under HKY and GTR models. Confidence was inferred by running 1,000 bootstrap replicates.

### cgMLST-based epidemiological analysis of S. mutans.

A minimum spanning tree was constructed based on our cgMLST scheme using 145 S. mutans isolates from 10 countries (China, *n* = 80; Brazil, *n* = 16; Denmark, *n* = 1; Germany, *n* = 2; Iceland, *n* = 5; Japan, *n* = 1; South Africa, *n* = 2; Turkey, *n* = 2, United Kingdom, *n* = 22, and Unite States, *n* = 14) via Perl 5.18.2 with a maximum length of 50 and opacity of 0.5. The whole-genome sequences of the 65 isolates from other countries are listed in Table S7 in the supplemental material.

10.1128/mSphere.00348-20.8 TABLE S6 Distribution of core genome sequence type (cgSTs) of the 297 isolates mentioned in this study. Download Table S6, XLSX file, 0.02 MB. Copyright © 2020 Liu et al. 2020 Liu et al. This content is distributed under the terms of the Creative Commons Attribution 4.0 International license.

10.1128/mSphere.00348-20.9 TABLE S7 Information including assembly, origin, release date, and cgST pertaining to the 65 isolates from other countries. Download Table S7, XLSX file, 0.01 MB. Copyright © 2020 Liu et al. 2020 Liu et al. This content is distributed under the terms of the Creative Commons Attribution 4.0 International license.

### Data availability.

All assembled reads of the 80 S. mutans clinical isolates were submitted to the National Center for Biotechnology Information (NCBI) under the BioProject accession numbers PRJNA598422 to PRJNA598424, PRJNA598427, PRJNA598791, PRJNA598793 to PRJNA598796, PRJNA598882, PRJNA598884, PRJNA598886, PRJNA598888, PRJNA598889, PRJNA600562, PRJNA600581, PRJNA600582, PRJNA600585, PRJNA600586, PRJNA600589, PRJNA600592 to PRJNA600599, PRJNA600601, PRJNA600602, PRJNA600684, PRJNA600687, PRJNA600688, PRJNA600690 to PRJNA600692, PRJNA600694 to PRJNA600697, PRJNA600699 to PRJNA600701, PRJNA600703 to PRJNA600706, PRJNA600708 to PRJNA600710, PRJNA601102, PRJNA601537 to PRJNA601539, PRJNA601543, PRJNA601544, PRJNA601546, PRJNA601547, PRJNA601549, PRJNA601552 to PRJNA601554, PRJNA601556, PRJNA601558, PRJNA601560, PRJNA601564, PRJNA601566, PRJNA601567, PRJNA601569, PRJNA601571 to PRJNA601574, PRJNA601577, PRJNA601578, PRJNA601594 to PRJNA601597, and PRJNA601599.
