# Supplementary figures and images for: A Core Genome Multilocus Sequence Typing Scheme for Streptococcus mutans
Source: mSphere. 2020 Jul 8;5(4):e00348-20. doi: 10.1128/mSphere.00348-20 (PMC7343978; doi:10.1128/mSphere.00348-20)

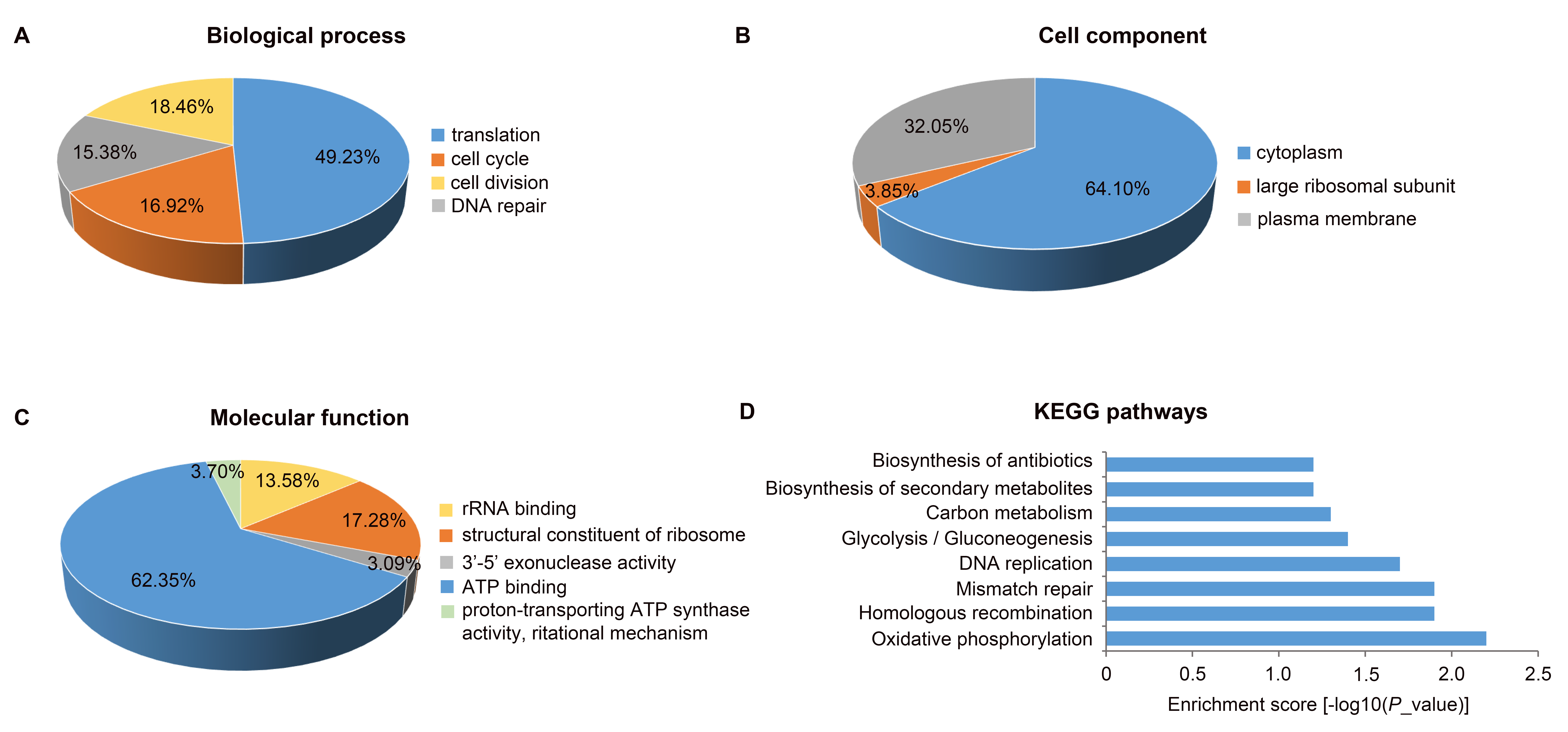

Supplement: FIG S1 [file mSphere.00348-20-sf001.tif]

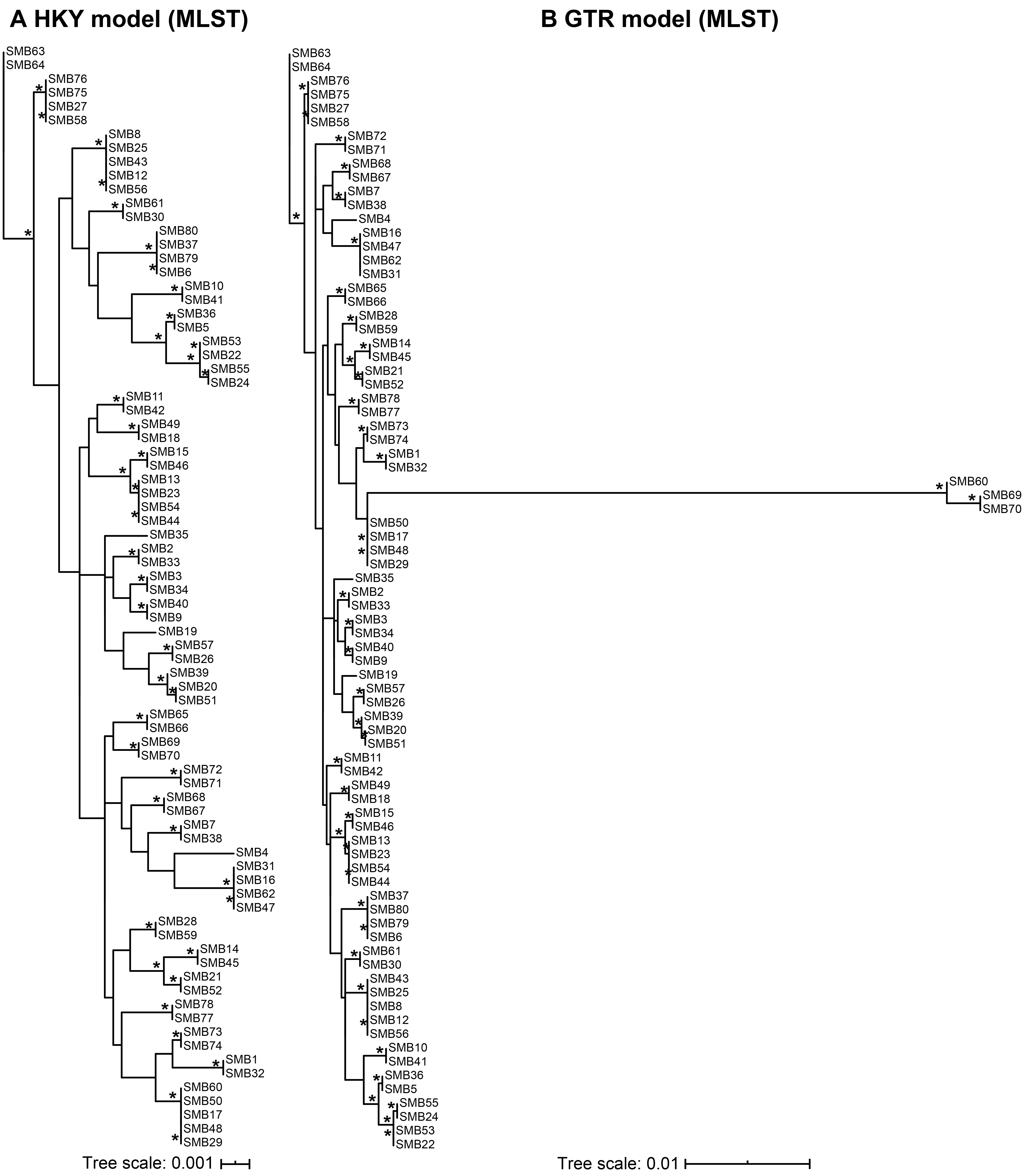

Supplement: FIG S2 [file mSphere.00348-20-sf002.tif]
